# Identification of *Enterobacter sakazakii *from closely related species: The use of Artificial Neural Networks in the analysis of biochemical and 16S rDNA data

**DOI:** 10.1186/1471-2180-6-28

**Published:** 2006-03-13

**Authors:** Carol Iversen, Lee Lancashire, Michael Waddington, Stephen Forsythe, Graham Ball

**Affiliations:** 1 The Nottingham Trent University, School of Biomedical and Natural Sciences, Clifton Campus, Clifton Lane, Nottingham, NG11 8NS, UK; 2 Loreus Ltd., Erasmus Darwin Building, College of Science and Technology, Nottingham Trent University, Clifton Lane, Nottingham, NG11 8NS, UK; 3 Accugenix, 223 Lake Drive, Newark, DE 19702, USA

## Abstract

**Background:**

*Enterobacter sakazakii *is an emergent pathogen associated with ingestion of infant formula and accurate identification is important in both industrial and clinical settings. Bacterial species can be difficult to accurately characterise from complex biochemical datasets and computer algorithms can potentially simplify the process.

**Results:**

Artificial Neural Networks were applied to biochemical and 16S rDNA data derived from 282 strains of *Enterobacteriaceae*, including 189 *E. sakazakii *isolates, in order to identify key characteristics which could improve the identification of *E. sakazakii*. The models developed resulted in a predictive performance for blind (validation) data of 99.3 % correct discrimination between *E. sakazakii *and closely related species for both phenotypic and genotypic data. Three main regions of the partial rDNA sequence were found to be key in discriminating the species. Comparison between *E. sakazakii *and other strains also constitutively positive for expression of the enzyme α-glucosidase resulted in a predictive performance of 98.7 % for 16S rDNA sequence data and 100% for phenotypic data.

**Conclusion:**

The computationally based methods developed here show a remarkable ability in reducing data dimensionality and complexity, in order to eliminate noise from the system in order to facilitate the speed and reliability of a potential strain identification system. Furthermore, the approaches described are also able to provide valuable information regarding the population structure and distribution of individual species thus providing the foundations for novel assays and diagnostic tests for rapid identification of pathogens.

## Background

*Enterobacter sakazakii *is an emergent pathogen associated with ingestion of infant formula milk that can lead to neonatal meningitis, necrotising enterocolitis and sepsis [1-5]. The International Commission for Microbiological Specifications for Foods [6] has ranked *E. sakazakii *as 'Severe hazard for restricted populations, life threatening or substantial chronic sequelae or long duration'. Therefore as there is no accepted gold standard methodology, the correct definition and identification of *E. sakazakii *is important for powdered infant formula manufacturers, as well as regulators, clinicians and epidemiologists.

In 1980, Farmer and co-workers [7] defined the species and described fifteen biogroups according to biochemical profiles. A defining characteristic has been activity of the α-glucosidase enzyme. Consequently selective, differential media incorporating chromogenic or fluorogenic α-glucosides such as the indolyl substrate 5-bromo-4-chloro-3-indolyl-α, D-glucopyranoside have been developed [8,9]. It has been reported that 100% of *E. sakazakii *(n = 129) were positive for α-glucosidase in comparison to 0% of other *Enterobacter *species (n = 97) [10]; however a small number of other *Enterobacteriaceae *test positive for this enzyme.

Recently 16S rDNA sequencing has revealed that commercial biochemical test kits identified more than one species as *'E. sakazakii' *[11], and that there are at least four genetically and biochemically distinct subgroups of *E. sakazakii*. In this study we applied Artificial Neural Networks (ANNs) [12-14] to biochemical and 16S rDNA data in order to identify key phenotypic characteristics and nucleotide sequences which could improve the identification of *E. sakazakii *in respect to, a) other *Enterobacteriaceae*, and b) non-*E. sakazakii *α-glucosidase positive *Enterobacteriaceae*.

ANNs are adaptive, non linear forms of Artificial Intelligence (AI) inspired by the way the human brain learns and processes information in order to solve specific problems, such as pattern recognition and classification problems. The multi-layer perceptron (MLP) ANN is a form of feed-forward ANN architecture which contains several layers, with each node in one layer being connected to every node in the next by a series of weighted links. When used with the back-propagation algorithm, this type of ANN learns in a fashion analogous to the way learning in the human brain is carried out, that is, by example. In humans, learning involves minor adjustments being made to the synaptic connections between neurons, in ANNs, learning is achieved by updating the weights that exist between the processing elements that constitute the network topology.

ANNs were applied to biochemical and 16S rDNA data derived from 282 strains of *Enterobacteriaceae*, including 189 *E. sakazakii *isolates, in order to identify key characteristics which could improve the identification of *E. sakazakii*. Results show that ANNs have the potential to identify key features from the data, both for biochemical tests and sequence data. These key features may then be used to form the basis of novel rapid identification systems, which have the ability to classify samples by strain and eliminate the risk of false positive and negative results.

## Results

**Figure 1:**
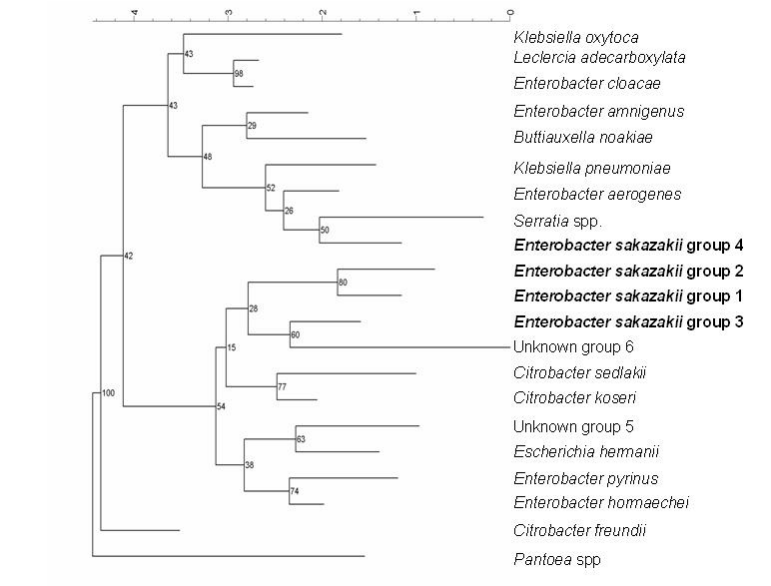
**Summary partial 16S rDNA sequence Neighbour Joining tree of *E. sakazakii *and related organisms**. Bootstraps were derived from 1000 replicates and the Jukes-Cantor correction was applied.

Food, clinical and environmental isolates of *E. sakazakii *were shown by 16S rDNA analysis to form four clusters. A summary of the main cluster groups is shown in Figure 1. The clusters that were positive for constitutive X-α-glucoside metabolism were the four *Enterobacter sakazakii *groups, *Buttiauxella noakiae*, and two clusters of *Enterobacteriaceae *(groups 5 and 6 in Figure 1) that could not be matched, either by genomic or biochemical profile, to any currently named species.

### Model development and classification analysis

A MLP ANN was used together with the back-propagation algorithm. Inputs to the network represented biochemical test results or sequence ID; two hidden nodes were used in the hidden layer for mathematical feature detection and a single output node was used to represent species class, with a class assignment of 1 representing *E. sakazakii *strains, and 2 representing all other strains. Models were developed utilising a random sample cross validation approach where 100 random training/test/validation sub-models were run and evaluated. This repeated random sampling guarantees that all samples are treated as blind data a number of times, to ensure model generality and to enable confidence intervals to be calculated for each sample. For each of the models a full analysis was conducted including sensitivity analysis to determine the importance ratio of each input. This process removed all of the inputs singularly from the model. The error of predictions was then measured for each of the inputs removed. The sensitivity ratio was then calculated based on the performance with and without the given input. The hypothesis here is that if a given input is important its removal will have strong negative effect on predictive performance. Therefore a sensitivity ratio greater than one indicates an input whose removal is detrimental for the model. Additionally, the analysis of predictive performance was performed to evaluate model accuracy, sensitivity and specificity, and assessment of the raw ANN predictions was conducted for the positioning of individuals within the population.

### Phenotypic data

**Figure 2:**
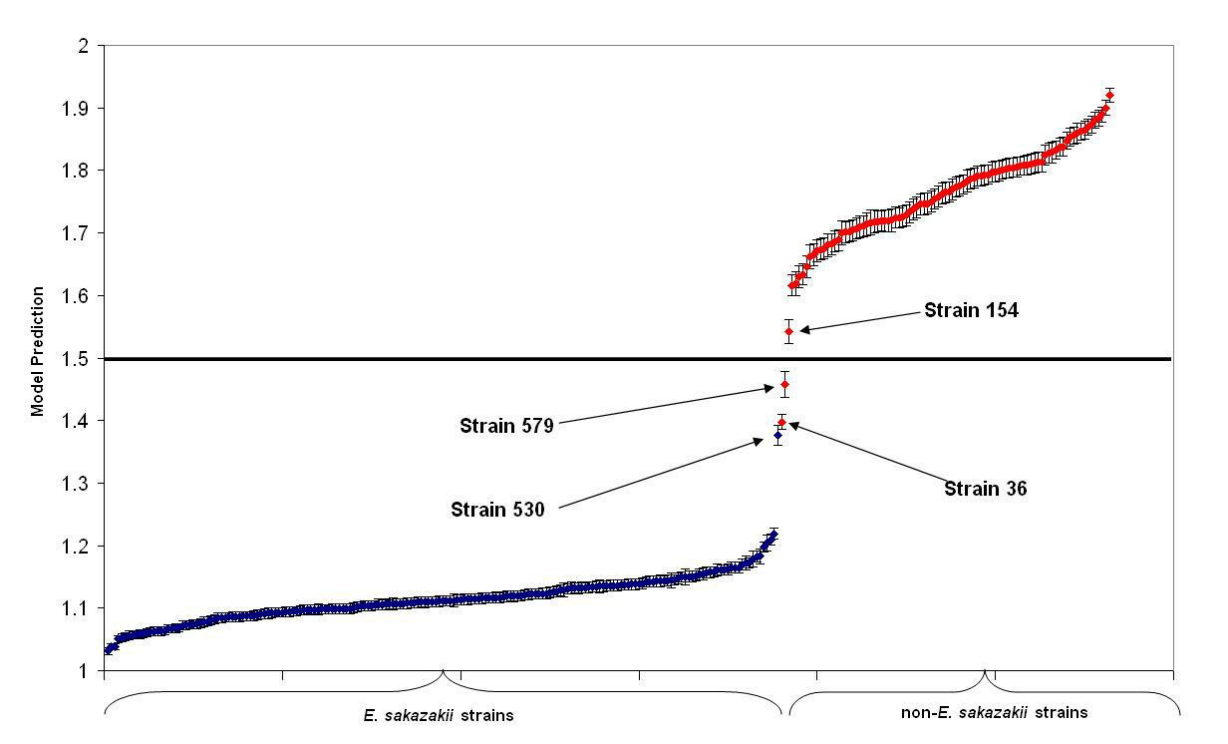
**Population distribution of samples from the biochemical test data**. Strains coloured blue represent *E. sakazakii *samples, whilst those in red represent non-*E. sakazakii*. The line at a predicted value of 1.5 represents the threshold for class prediction. Error bars indicate 95 % confidence intervals, and labelled samples highlight those which were either misclassified or close to being so.

Using the phenotypic data, the models developed resulted in a predictive performance for blind (validation) data of 99.3 % (sensitivity of 100 % and specificity of 97.6 %) correct discrimination between *E. sakazakii *and closely related species. The population distribution was also examined by plotting the individual predictions from the ANN models (Figure 2). A model prediction of one indicates a sample is *E. sakazakii *whilst a two is indicative of another species, so as this value increases from one to two, the more characteristic a sample is of non-*E. sakazakii *origin. This distribution shows the variation that is present not only between the same strains, but also across species, which is why correct identification of pathogens can often be extremely difficult, with strains having the potential for frequent mutation and change.

### 16S rDNA sequence data

**Figure 3:**
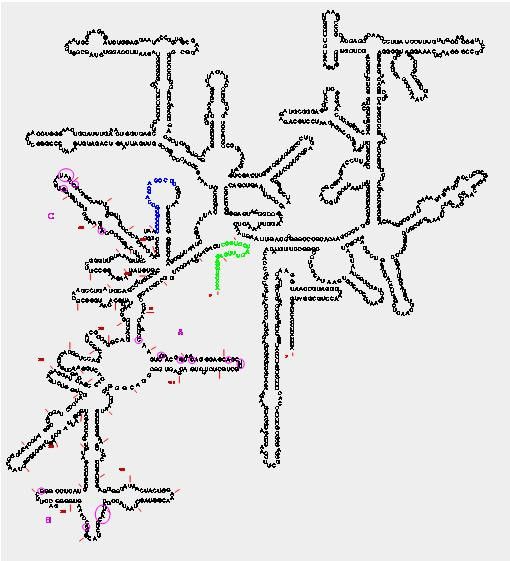
**Secondary structure: small subunit ribosomal RNA of *E. sakazakii *NCTC 11467**. Nucleotides in green and blue represent primer sequence targets. Pink circles denote regions of importance as determined by Artificial Neural Networks. Nucleotide numbering follows the Reference Numbering System used for *E. coli *J01695 [15]. Every 10th nucleotide is marked with a red tick mark and every 50th nucleotide is numbered. (Structure courtesy of Doug Smith, Accugenix, DE, USA).

The analysis was also repeated using 16S rDNA sequence data to identify any areas of the sequence that could potentially be used to differentiate between the different species. The models developed produced predictive performances for blind (validation) data to an accuracy of once again, 99.3 % (with sensitivity and specificity values of 99.5 and 98.9 % respectively) of samples correctly identified as *E. sakazakii *or other species. There were three main regions of the sequence which were key in discriminating between the species. These regions all occur amongst regions that vary structurally among domains (see secondary structure Figure 3). Table 1 shows the 20 nucleotides with greatest relative importance and it is evident that they all appear to be derived from these focal positions in the sequence.

### Classification of α-glucosidase positive strains

Furthermore, the study was expanded in order to elucidate whether the ANNs could be used to differentiate between *E. sakazakii *and a number of other *Enterobacteriaceae *which test positive for constitutive metabolism of X-α-glucoside. The same approach was used as above, with both phenotypic tests and 16S rDNA sequencing used as inputs in the ANN models. Once again analysis using the ANN based approach proved to be extremely successful. Using the 16S rDNA sequence data as inputs, the predictive performance of the ANN models was 98.7 % (92.9 % sensitivity, and 100 % specificity). This improved further still when the biochemical test data results were used as inputs into the model. Here, 100 % of the strains were correctly predicted into their respective classes, further highlighting the capabilities of ANN modelling in bacterial identification, which could potentially reduce the risk of false positive identification. The most relevant biochemical tests are summarised in Table 2, showing percent positive strains for *E. sakazakii *as well as other α-glucosidase positive and negative *Enterobacteriaceae*.

## Discussion

Models have been developed to identify (i) key biochemical tests and (ii) important areas of the DNA sequence which can be used in the accurate discrimination of *E. sakazakii *from other closely related species. Furthermore, the study was expanded to differentiate between *E. sakazakii *strains and other α-glucosidase positive *Enterobacteriaceae*. To date methods for the isolation and identification of *E. sakazakii *have used the α-glucosidase reaction and production of yellow pigment as presumptive differentiating characteristics. However these methods can result in presumptive false positives due to groups of as yet undefined non-*E. sakazakii Enterobacteriaceae *which are also positive for both of these characteristics. Use of yellow pigment as a defining characteristic can also result in false negatives due to the occurrence of non-pigmented *E. sakazakii *and the occasional transient nature of this trait. While there is no single test that can be used to differentiate *E. sakazakii *from other species we identified biochemical profiles that may help to improve the likelihood of correct species identification.

Deriving a population distribution (Figure 2) from the analysis of the influence of biochemical tests in sample classification showed samples to appear in distinct clusters. This supports the interpretation of the partial 16S rDNA clustering (Figure 1). The ANN model incorrectly identified two of the non-*E. sakazakii *strains as being *E. sakazakii*. The identities of these strains are highlighted in Figure 2, and these samples may provide a basis for further studies because they are being incorrectly classified as a result of them displaying characteristics of both of the two groups, but are being determined to be more related to the *E. sakazakii *group. Alternatively, since the 16S identification is based on differences between a number of nucleotide bases, the combinations of these is different for different species. The ANN models search for common elements of these bases, which are consistently represented in each class, and classifies based on these commonalities. Considering this, together with the incorrectly identified non- *E. sakazakii *strains, leads to the view that there may not be one base, or a series of bases, that are unique to *E. sakazakii*, and in some strains, such as those incorrectly identified by the model, common elements exist between *E. sakazakii*, and other strains.

Results from the analysis of the 16S rDNA data indicate that the key inputs identified were present in three distinct areas of the sequence and these areas were subsequently all regions that varied structurally among domains (Figure 3).

## Conclusion

ANNs display their potential use in reducing model dimensionality and complexity, in order to facilitate the speed and reliability of a potential strain identification system. These methods are also able to provide valuable information regarding the population structure and distribution of individual species. These technologies may provide the foundations for novel assays and diagnostic tests for rapid identification of pathogens, and subsequently reducing the risk of incorrect diagnosis due to the occurrence of false positive and negative test results.

## Methods

Genotypic and phenotypic data was collected for 282 strains of *Enterobacteriaceae*, including 189 *E. sakazakii *isolates and 39 other α-glucosidase positive strains. Strains were from diverse food, clinical and environmental sources worldwide. Clinical isolates were from cases occurring over the last 25 years. At least one original strain from each of the biogroups described when the *E. sakazakii *species was designated were included [7].

### Phenotypic data

Biochemical characteristics were derived from commercial test kits (API 20E and ID32E, bioMérieux UK Ltd.; Biolog GN2, Biolog, CA; and Microbact 24E, Oxoid UK Ltd.) and conventional manual tests as per standard protocols. Tests were performed in triplicate on separate days. Motility was determined at 37°C after 24 h and 48 h using motility medium (tryptose 10 g l^-1^, NaCl 5 g l^-1^, agar 5 g l^-1^, pH 7.2 ± 0.2. Acid production from carbohydrates was tested in phenol red broth base (10 g l^-1 ^peptone, 1 g l^-1 ^yeast extract, 5 g l^-1 ^NaCl, 0.018 g l^-1 ^phenol red) with addition of filter-sterilized carbohydrate solution (final concentration 0.5%). Gas production was determined by collection in Durham tubes. The methyl red test was performed at 48 h on cultures grown in MR-VP broth (VWR, 1.05712.0500). The Voges-Proskauer test was performed at 24 h by addition of 40% potassium hydroxide in water and 5% 1-naphthol in ethanol to cultures grown in MR-VP broth. Indole production was measured at 24 h by addition of Kovacs reagent (5 g p-dimethylaminobenzaldehyde, 25 ml HCl, 75 ml pentanol-1-ol) or James Reagent (70542 bioMérieux) to cultures grown in Peptone Water (CM0009 Oxoid Ltd). Nitrate reduction was measured by addition of 1% sulphanilamide in 1 M HCl and 0.02% N-1 naphthylene diamine HCl in water. Zinc dust was added to negative tubes to confirm the presence of unreduced nitrate. Constitutive metabolism of X-α-glucoside was determined by formation of blue-green colonies on media containing 5-bromo-4-chloro-3-indolyl-α, D-glucopyranoside (Chromogenic Enterobacter sakazakii medium (DFI formulation) CM1055, Oxoid Ltd.; and ESIA, AES Laboratoire, France).

### Comparative 16S rDNA sequencing

This was performed by Accugenix (Newark, DE, USA) using the MicroSeq™ 500 16S rDNA Bacterial Sequencing Kit (Applied Biosystems). DNA was prepared for PCR by quick-heat lysis by removing one colony into a tube of PrepMan Ultra™ (Applied Biosystems) and placed at 99°C for 10 min. Two microlitres of genomic DNA was amplified in 50 μl of a master mixture consisting of 0.4 μM TGGAGAGTTTGATCCTGGCTCAG and TACCGCGGCTGCTGGCAC primers, 200 mM deoxynucleoside triphosphates, PCR buffer, 0.3 U of AmpliTaq DNA polymerase, and 10% glycerol. PCR conditions were 95°C for 10 min; 30 cycles each of 95°C for 30 s, 60°C for 30 s, and 72°C for 45 s; and a final step at 72°C for 10 min. Purification of the PCR product to remove excess primers and nucleotides was performed using Montage SEQ_96 _filter plates (Millipore). Cycle sequencing was performed with the sequencing module, and after removal of excess dyes using Montage SEQ_96 _filter plates (Millipore), the labelled extension products were separated on an ABI 3100 16 capillary genetic analyzer (Applied Biosystems). Partial sequencing was performed for all isolates, the length of the partial rDNA was 528 nucleotides, and in addition the full sequence for the *E. sakazakii *type strain (NCTC 11467) was obtained.

The data was analysed using Bionumerics (Applied Maths, Belgium) to construct Neighbour Joining trees, bootstraps were derived from 1000 replicates and the Jukes-Cantor correction applied.

The full 16S sequence was used for the representation of the secondary structure of the small subunit ribosomal RNA of E. sakazakii NCTC 11467. Nucleotide numbering follows the Reference Numbering System used for E. coli J01695 [15].

## List of abbreviations

AI Artificial Intelligence

ANN Artificial Neural Network

MLP Multi-Layer Perceptron

## Authors' contributions

CI performed the biochemical characterizations, collated the test data and wrote the biochemical methods section and text relevant to *E. sakazakii*. LL co-developed and performed the computational analyses for the study, and drafted the manuscript. MW provided the 16S sequencing and wrote the 16S sequencing methods section. SF co-ordinated and managed the project. GB co-developed the analysis methods and co-ordinated the project. All authors read and approved the final manuscript.

## Figures and Tables

**Table 1:** Top 20 nucleotides involved in classification from partial 16S rDNA data. Regions shown in this table are highlighted in Figure 3.

REGION		
A	B	C
58	180	448
64	181	462
69	182	467
72	183	468
81	192	469
84	211	471
85		

**Table 2:** Biochemical tests for the differentiation of *E. sakazakii*.

Biochemical Tests	*E. sakazakii *(n = 189)	other α-glucosidase positive strains (n = 39)	other Enterobacteriaceae (n = 54)
α-glucosidase	100 *	100	0
Arginine dehydrogenase	97	13	67
Citrate	99	15	80
D-saccharic acid	0	23	33
Dulcitol	8	80	28
glucose-1-phosphate	0	59	83
glucose-6-phosphate	0	46	82
Lipase	96	44	4
Methyl Red	5	95	57
Ornithine decarboxylase	91	0	74
Pyruvate	3	92	50
Raffinose	100	15	63
Sucrose	100	21	47
Voges Proskauer	96	0	44
Yellow pigment	98	90	28

* denotes percent strains from the data set which were positive for the test
